# PTK6/BRK is expressed in the normal mammary gland and activated at the plasma membrane in breast tumors

**DOI:** 10.18632/oncotarget.2153

**Published:** 2014-06-30

**Authors:** Maoyu Peng, Rajyasree Emmadi, Zebin Wang, Elizabeth L. Wiley, Peter H. Gann, Seema A. Khan, Nilanjana Banerji, William McDonald, Szilard Asztalos, Thao N.D. Pham, Debra A. Tonetti, Angela L. Tyner

**Affiliations:** ^1^ Departments of Biochemistry and Molecular Genetics, University of Illinois at Chicago, Chicago IL; ^2^ Department of Biopharmaceutical Sciences University of Illinois at Chicago, Chicago IL; ^3^ Department of Pathology, University of Illinois at Chicago, Chicago IL; ^4^ Department of Surgery, Northwestern Feinberg School of Medicine, Chicago, IL; ^5^ Allina Health, Minneapolis, MN, USA

**Keywords:** PTK6, BRK, tyrosine kinase, breast cancer

## Abstract

Protein Tyrosine kinase 6 (PTK6/BRK) is overexpressed in the majority of human breast tumors and breast tumor cell lines. It is also expressed in normal epithelial linings of the gastrointestinal tract, skin, and prostate. To date, expression of PTK6 has not been extensively examined in the normal human mammary gland. We detected PTK6 mRNA and protein expression in the immortalized normal MCF-10A human mammary gland epithelial cell line, and examined PTK6 expression and activation in a normal human breast tissue microarray, as well as in human breast tumors. Phosphorylation of tyrosine residue 342 in the PTK6 activation loop corresponds with its activation. Similar to findings in the prostate, we detect nuclear and cytoplasmic PTK6 in normal mammary gland epithelial cells, but no phosphorylation of tyrosine residue 342. However, in human breast tumors, striking PTK6 expression and phosphorylation of tyrosine 342 is observed at the plasma membrane. PTK6 is expressed in the normal human mammary gland, but does not appear to be active and may have kinase-independent functions that are distinct from its cancer promoting activities at the membrane. Understanding consequences of PTK6 activation at the plasma membrane may have implications for developing novel targeted therapies against this kinase.

## INTRODUCTION

Protein tyrosine kinase 6 (PTK6, also called BRK) is a SRC-related intracellular tyrosine kinase expressed in normal epithelia and cancer. Like SRC, it contains amino terminal SH3 and SH2 domains, but PTK6 is not palmitoylated or myristoylated and displays flexibility in its intracellular localization [1]. PTK6 was identified in human metastatic breast cancer [2], and is overexpressed in the majority of human breast cancers and in most breast tumor cell lines [3-5].

Recently, PTK6 intracellular localization has been highlighted as an important regulator of its signaling in the prostate [6]. PTK6 is expressed in normal prostate epithelial cells where it is largely localized to the nuclei, but in prostate cancers PTK6 nuclear localization is lost [7]. Targeting PTK6 into the nucleus of prostate cancer cells in vitro negatively regulates growth [8]. In human and mouse prostate cancer cells, PTK6 is activated at the cell plasma membrane [9-11]. A number of membrane associated PTK6 substrates, including Paxillin [12], EGFR [13], IGF-1R [14], p130Cas [9], and focal adhesion kinase [10] have been identified. Targeting PTK6 to the plasma membrane by addition of a palmitoylation/myristoylation signal promoted the epithelial mesenchymal transition and enhanced growth and metastasis of prostate cancer xenograft tumors [11].

Several studies have suggested that PTK6 is a potential therapeutic target in breast cancer. It promotes signaling by a number of receptor tyrosine kinases including members of the ERBB receptor family, MET, and IGF-1R (reviewed in [15, 16]). PTK6 cooperates with ERBB2 to promote oncogenic signaling [17-20]. In addition, PTK6 confers cancer cell resistance to anoikis [10, 21] and knockdown of PTK6 increases sensitivity of cancer cells to different therapeutic agents [13, 22]. Transgene expression of PTK6 in the mouse mammary gland enhances mammary gland tumorigenesis in vivo [23, 24].

In normal epithelia, highest levels of PTK6 expression are detected in the linings of the gastrointestinal tract and skin [25]. Disruption of the *Ptk6* gene led to impaired differentiation and increased growth in the mouse small intestine [26]. PTK6 also plays a positive role in keratinocyte differentiation [27, 28]. In human and mouse prostate, nuclear PTK6 expression is associated with differentiated glands [7, 11].

Significant levels of PTK6 expression are detected in most human breast tumors and breast cancer cell lines. We detected PTK6 in the nontransformed MCF-10A mammary gland epithelial cell line, leading us to examine its expression in a normal human mammary gland tissue array. Interestingly, total PTK6, but not active PTK6, was detected in epithelial cells of normal glands. Like previously reported for the prostate, PTK6 protein was frequently localized to nuclei in normal cells. Staining of breast tumor tissue microarrays (TMAs) demonstrated increased levels of PTK6 expression that was often in the active form at the plasma membrane in tumor cells. This is the first report of PTK6 expression in the human normal mammary gland. Activation of PTK6 at the cell membrane highlights the need for development of strategies to target membrane specific functions of PTK6 in cancer.

## RESULTS

### PTK6 is expressed in normal mammary gland epithelia

**Figure 1:**
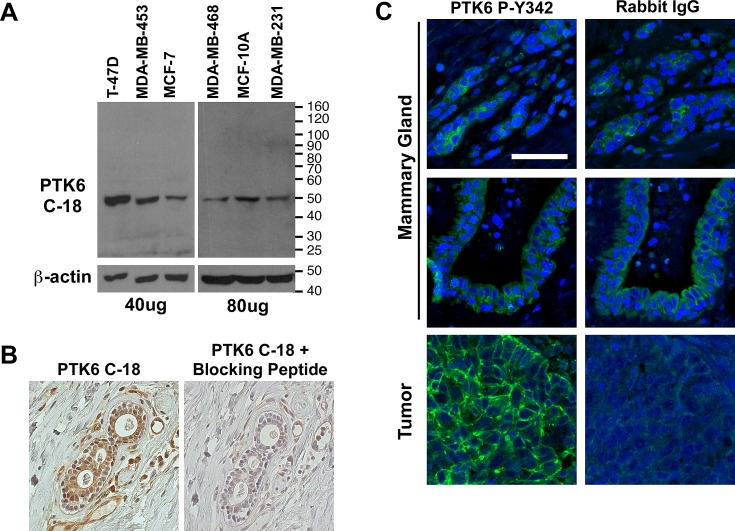
Controls were performed to confirm the specificity of PTK6 antibody for detection of PTK6 in nontransformed cells and tissues (A) Total cell lysates from several human breast cell lines were probed with a 1:2000 dilution of PTK6 C-18 antibody (Santa Cruz), and the entire membrane is shown. A specific 50 kDa band corresponding to PTK6 is detected in all samples. (B) Specificity of the PTK6 C-18 antibody for immunohistochemistry was confirmed using serial sections of normal human mammary gland and PTK6 C-18 antibody plus and minus pre-incubation with the PTK6 immunogenic peptide. Blocking with PTK6 peptide eliminates detection of the cytoplasmic and nuclear staining obtained with the C-18 antibody. (C) P-Y342 PTK6 was detected only in human breast tumors. Normal mammary gland and tumors were stained with PTK6 P-Y342 antibody or rabbit IgG as a control. The same gain time for FITC was used when taking both PTK6 and IgG pictures (scale bar = 50 μm).

PTK6 is expressed in breast cancer cell lines representing different molecular subtypes of breast cancer. We detected both *PTK6* mRNA and protein expression in all of breast cancer cells lines that we examined, as well as in the nontransformed MCF-10A human mammary gland epithelial cell line (Figs. 1A, S1A-C). Expression of an alternatively spliced *ALT-PTK6* transcript that lacks exon 2 and encodes a shorter 15 kDa protein containing the SH3 domain and a unique proline rich carboxy terminus, as well as transcripts encoding the full length PTK6 was previously reported in the T-47D breast cancer cell line [29] and multiple human prostate and colon cell lines [30]. We detected *ALT-PTK6* transcripts in all breast cancer cell lines analyzed by semi-quantitative PCR (Figure S1C), although the ratio of full length *PTK6* to *ALT-PTK6* varied from cell line to cell line. Interestingly the level of expression of the *ALT-PTK6* transcript was extremely low in MCF10A cells compared with the breast cancer cell lines, although it could be clearly detected with increased cDNA input. The function of ALT-PTK6 is still poorly understood, although it may compete with full-length PTK6 [30]. Unfortunately, we have not identified antibodies that detect the endogenous human ALT-PTK6 protein.

Detecting PTK6 expression in the MCF-10A cell line and recognizing that PTK6 is also expressed in normal epithelia of the gastrointestinal tract, skin and prostate, led us to re-examine PTK6 expression in the normal mammary gland using a normal mammary gland tissue microarray (TMA). Prior to examining PTK6 protein expression, the specificity of commercially available PTK6 antibodies was evaluated by immunoblotting and immunohistochemistry. The Santa Cruz Biotechnology BRK C-18 polyclonal (Fig. 1A) and BRK G-6 monoclonal antibodies (Fig. S1B) both recognize a specific PTK6 band in breast tumor cell lysates. PTK6 protein expression levels correlate well with expression of its mRNA (Fig. S1A). We found that of these two antibodies, the C-18 antibody produced the best signal in mammary gland tissue in immunohistochemistry studies. Specificity of the C-18 antibody immunohistochemistry signal was confirmed in a competition assay performed with the immunogenic PTK6/BRK peptide used for antibody production. Preincubation of the peptide with the C-18 antibody efficiently eliminated the PTK6 signal in the normal mammary gland (Fig. 1B).

Phosphorylation of tyrosine residue 342 (P-Y342) in the PTK6 activation loop promotes its activation [31], and may serve as a marker for increased PTK6 activity. PTK6 tyrosine residue 342 corresponds to human SRC tyrosine residue 419, but with the exception of the tyrosine, the peptide sequence immediately surrounding the tyrosine residues is not highly conserved. The specificity of an antibody available from Millipore that detects PTK6 P-Y342 was recently demonstrated [11]. In addition, we have found that the PTK6 P-Y342 antibody recognizes only active PTK6 but not kinase-defective PTK6 in expression studies, and we can detect PTK6 P-Y342 immunoreactivity in mammary gland tumors that formed in wild type but not *Ptk6*−/− mice, further indicating antibody specificity (data not shown). We did not detect specific PTK6 P-Y342 immunoreactivity in normal human mammary gland tissue. The signal in normal tissue was equivalent to the IgG control (Fig. 1C). However, striking membrane localization of PTK6 P-Y342 was detected in human mammary gland tumors (Fig. 1C).

**Figure 2:**
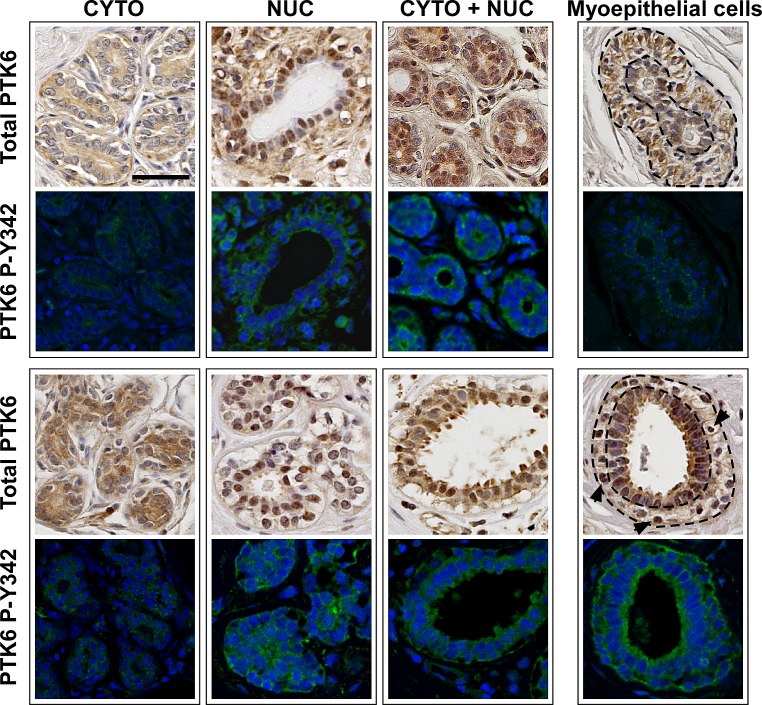
PTK6 is expressed in normal human mammary gland but it is not phosphorylated at tyrosine residue 342 Immunohistochemistry and immunofluorescence assays were performed on serial sections of human mammary gland tissue microarray. The TMA cores contained normal mammary glands obtained from breast reduction or benign biopsy. Antibodies against total human PTK6 and P-Y342 PTK6 were used to detect endogenous PTK6. Weak to medium levels of PTK6 were detected in luminal cells of most cores, and expression was cytoplasmic, nuclear or in a mixed pattern. Weak PTK6 expression was also detected in myoepithelial cells, and its subcellular localization varied, with it being predominately cytoplasmic (upper right corner, outlined by dashed lines) and in a few cases in the nuclei of vacuolated myoepithelial cells (arrows, lower right corner, outlined by dashed lines). However, active PTK6 P-Y342 (FITC green) was not detected in the normal mammary glands. Signal shown is equivalent to the IgG signal in Figure 1 (scale bar = 50 μm).

A TMA of normal mammary gland tissue from reduction mammoplasty and benign breast biopsies from women 18 to 45 years of age [32] was stained for total and active P-Y342 PTK6 (Fig. 2). Corresponding sections from individual patients are shown in the boxed regions. PTK6 protein expression was detected in the cytoplasm and/or nucleus of normal mammary gland epithelial cells. All groups of samples examined were positive for cytoplasmic PTK6 staining, with two-thirds of these also exhibiting nuclear expression (Table 1). PTK6 expression was also detected in myoepithelial cells.

**Table 1:** PTK6 expression in normal human mammary gland PTK6 protein expression was examined in 76 cores of mammary gland tissue from 27 patients. Results are summarized for each patient

Cell type and subcellular localization		PTK6 Positive	Signal strength			
			0	1+	2+	3+
Luminal	Cytoplasm	27 (100%)	0 (0%)	13 (48.1%)	13 (48.1%)	1 (3.7%)
	Nucleus	17 (63.0%)	10 (37.0%)	5 (18.5%)	12 (44.4%)	0 (0%)
Myoepithelial	Cytoplasm	26 (96.3%)	1 (3.7%)	22 (81.5%)	4 (14.8%)	0 (0%)
	Nucleus	5 (18.5%)	22 (81.5%)	3 (11.1%)	2 (7.4%)	0 (0%)

### Active PTK6 P-Y342 is enriched at the plasma membrane in mammary gland tumors

**Figure 3:**
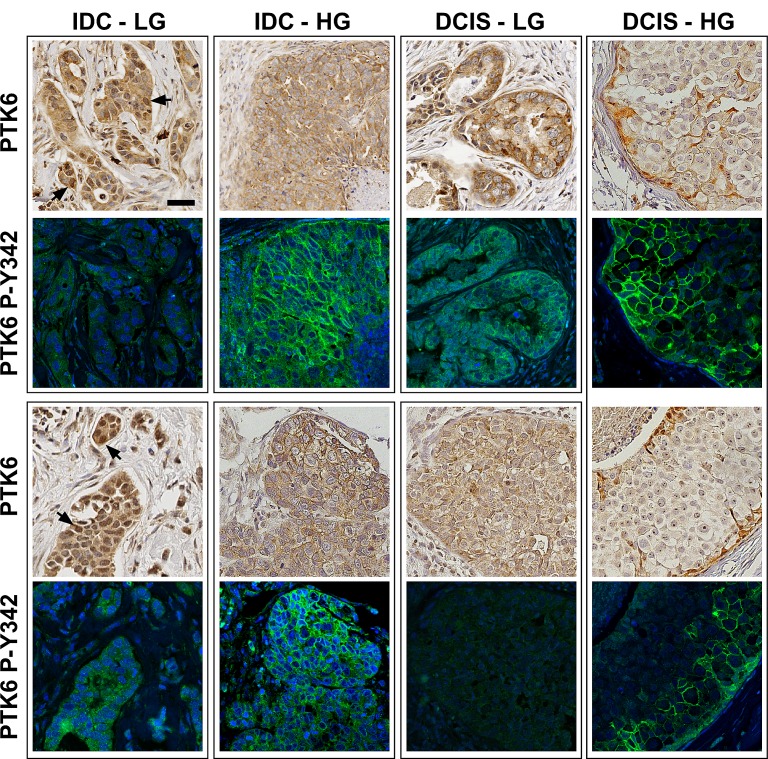
Active P-Y342 PTK6 is localized at the plasma membrane in high-grade ductal carcinomas Immunohistochemistry and immunofluorescence assays were performed on breast tumor TMA slides using the same conditions as normal breast TMA. Medium to strong levels of total PTK6 protein immunoreactivity were detected in breast tumor tissues (IDC: invasive ductal carcinoma. DCIS: ductal carcinoma in situ. LG: low-grade. HG: high-grade). Total PTK6 and P-Y342 PTK6 were shown in boxed pairs, with each box representing a different patient, and two patients are shown for each tumor type (upper and lower panels) with the exception of the DCIS-HG where two different cores from the same patient are shown. Although some nuclear staining of total PTK6 can be found in the low-grade carcinoma (arrows), it was absent from most of the high-grade carcinomas and PTK6 appears to be in the cytoplasm and at the membrane. Similar expression patterns are observed in ductal carcinoma in situ. In the low-grade ductal carcinomas, the P-Y342 signal is low, but in the high-grade invasive ductal carcinomas, strong P-Y342 PTK6 signal was detected at the membrane of tumors cells that have breached basement membrane. In the DCIS, active PTK6 is localized at the membrane of those cells close to the basement membrane (scale bar = 50 μm). Breast tumor subtypes include luminal B (both IDC-LG panels, and all panels of DCIS) and triple negative (IDC-HG, both panels)

Although it is well established that PTK6 is expressed in most human breast tumors, the activation status of PTK6 in breast tumors has not been examined. Immunohistochemistry and immunofluorescence were performed on a breast tumor TMA containing 131 cores from 45 patients (three different cores from each patient), using the same conditions as for the normal breast TMA. 125 cores from 44 patients contained enough tissue for total and P-Y342 PTK6 staining and corresponding cores for ER/PR/HER2 readings. PTK6 signal strength was scored from 0 to 3 by two board certified pathologists (R. Emmadi and E. L. Wiley) (Fig. S2). Total PTK6 was detected in most patients, who exhibited medium (2+) to strong (3+) signal strength (Table 2) (Fig. 3). Tumor subcategories were determined by analyzing corresponding H & E stained sections. The majority of the samples were invasive ductal carcinomas (IDC), both high and low-grade, with a few ductal carcinomas in situ (DCIS), lobular carcinomas in situ (LCIS) and mammary glands with micro invasion. The most intense cytoplasmic PTK6 staining was detected in invasive ductal carcinomas, while the highest nuclear PTK6 signals were detected in lobular carcinoma in situ. Although some nuclear staining of PTK6 was found in low-grade carcinomas (Fig. 3, arrows), it was absent from most high-grade carcinomas. Nuclear/cytoplasmic PTK6 was detected in low-grade tumors and became more cytoplasmic and membrane associated in higher grade tumors (Fig. 3).

**Table 2:** PTK6 expression, activation, and localization in human breast tumors PTK6 expression was examined in 125 cores from 44 patients. If a patient had variable PTK6 staining between cores, the highest value is indicated per patient. Subcellular localization patterns of PTK6 (membrane, cytoplasm and nucleus) are not always mutually exclusive

Antibodies and intracellular localization		PTK6 Positive	Signal strength			
			0	1+	2+	3+
Total PTK6	Cytoplasm	43 (97.7%)	1 (2.3%)	13 (29.5%)	28 (63.6%)	2 (4.5%)
	Nucleus	20 (45.4%)	24 (54.5%)	7 (15.9%)	12 (27.3%)	1 (2.3%)
PTK6 P-Y342	Membrane	18 (40.9%)	26 (59.1%)	12 (27.2%)	4 (9.1%)	2 (4.5%)
	Cytoplasm	14 (31.8%)	30 (68.2%)	11 (25.0%)	3 (6.8%)	0 (0%)
	Nucleus	5 (11.4%)	39 (88.6%)	4 (9.1%)	1 (2.3%)	0 (0%)

Interestingly, overexpression of PTK6 did not necessarily correlate with increased levels of PTK6 P-Y342. While more than 90% of patients overexpressed PTK6, only half of the patients were positive for active P-Y342 PTK6. Membrane localized active PTK6 was detected in about 40% of the patients, and the signals ranged from weak (27.2%) to strong (4.5%) (Table 2). Active cytoplasmic PTK6 was detected in 31.8% of the patients, but the signal was not as strong as the membrane staining. Active PTK6 appeared to correlate with grade of tumor; in low-grade ductal carcinomas, the P-Y342 signal was low to undetectable, but in the high-grade invasive ductal carcinomas, a strong P-Y342 PTK6 signal was detected at the membrane of tumors cells that had breached the basement membrane (Fig 3). In two out of three high-grade DCIS, active PTK6 P-Y342 was localized at the membrane (Fig. 3, FITC), while little or no PTK6 PY-342 was detected in three different low-grade DCIS samples.

**Figure 4:**
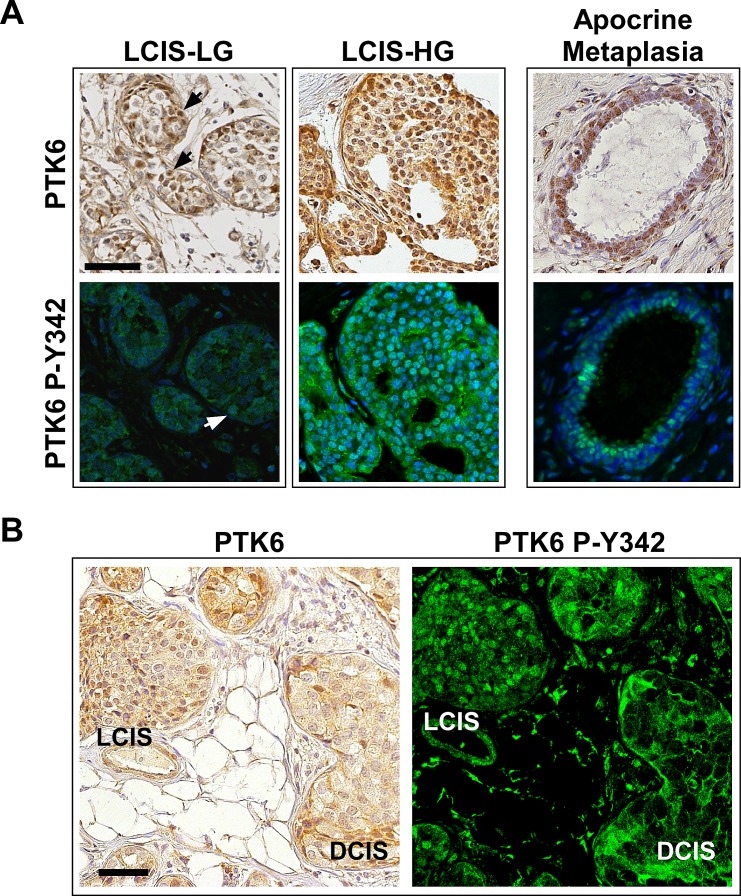
Active P-Y342 PTK6 is localized in the nucleus in lobular carcinomas and apocrine metaplasia PTK6 expression was detected in lobular carcinoma in situ. Unlike ductal carcinomas, PTK6 was mostly nuclear localized in the lobular carcinomas. (A) Left: Medium to strong levels of total and P-Y342 PTK6 were found in the nucleus of lobular carcinoma in situ of both low (arrows) and high-grade, while the membrane and cytoplasmic staining is very low or undetectable. Right: Total and active PTK6 localized in the nuclei of the luminal cells in apocrine metaplasia. (B) A pair of tumor cores containing both LCIS and DCIS was stained by total PTK6 (left, DAB) and PTK6 P-Y342 antibodies (right, FITC). Different PTK6 subcellular localization was detected: PTK6 was localized in the nucleus in LCIS and at the plasma membrane in DCIS (scale bar = 50 μm). Breast tumor subtypes: Luminal B (LCIS-LG) and luminal A (LCIS-HG and LCIS/DCIS).

Unlike in the ductal carcinomas, PTK6 was nuclear in the LCIS that we examined. As shown in Figure 4A, medium to strong levels of total PTK6 were detected in nuclei of both a low and a high-grade LCIS, while membrane and cytoplasmic PTK6 levels are very low or undetectable. Just like total PTK6, active PTK6 accumulated in cell nuclei, with signals ranging from very weak (white arrow) in the low-grade LCIS to strong in the high-grade LCIS (Fig. 4A). Most high-grade LCIS cells were positive for nuclear PTK6, but some PTK6 negative cells were also present. The difference between LCIS and DCIS is shown in a representative core containing both LCIS and DCIS (Figure 4B): total and active PTK6 are nuclear in LCIS, but at the membrane in DCIS. Increased numbers of breast tumor samples will need to be analyzed to determine the statistical significance of the intracellular localization patterns of total and active PTK6 in high versus low grade LCIS and DCIS.

**Figure 5:**
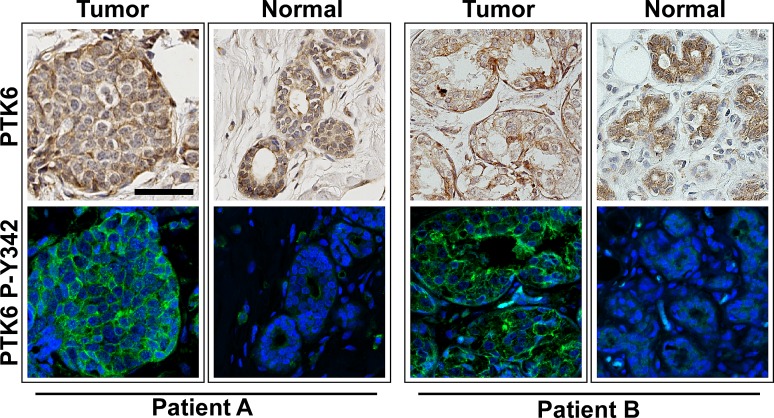
PTK6 P-Y342 is detected in the tumor but not in normal regions in biopsies from the same patient Three TMA cores were available from each patient, and some contained both malignant and benign tissue. When comparing the PTK6 expression patterns in tumor and nonmalignant tissue from same patient, we found that although the total PTK6 level may not change significantly, active PTK6 is only detectable in the tumor tissue but not in the normal looking glands. Tissue cores from two different patients are shown (scale bar = 50 μm). Tumors from both patients were classified as luminal A.

The tumor TMAs contained three cores from each patient, and some of these contained both malignant and benign tissue. When analyzing PTK6 expression in normal and tumor tissue from the same patient, we found that active P-Y342 PTK6 is only detectable in the tumor tissue but not in normal glands, although total PTK6 levels may not change much from normal to tumor tissue (Figure 5). PTK6 activation may be more significant than total PTK6 expression when evaluating breast tumor pathology.

### High expression of PTK6 correlates with poor survival outcome

**Figure 6:**
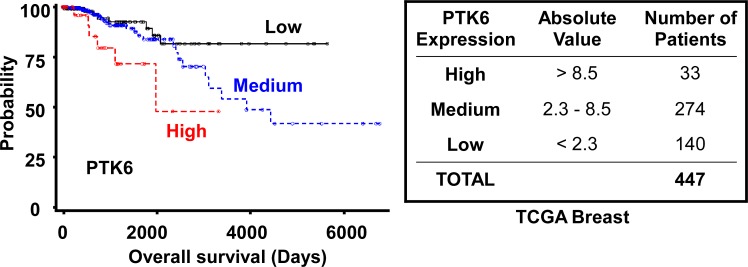
High *PTK6* mRNA expression is correlated with poor survival outcome *PTK6* expression data in the TCGA Breast dataset available from Oncomine were divided into “high” > 8.5, “medium” 2.3 - 8.5, and “low” < 2.3 groups. Survival probability was estimated using the Kaplan-Meier method and differences among three groups were tested using the log-rank test and the *P*-value (0.0107 by Log-Rank, 0.0241 by Wilcoxon).

The TCGA Breast dataset [33] was extracted from Oncomine and the relationship between survival and *PTK6* expression was examined (Figure 6). *PTK6* mRNA levels were divided into “high” > 8.5, “medium” 2.3 - 8.5, and “low” < 2.3 categories, and the number of patients in each category are listed in Figure 6. The survival curve was estimated using the Kaplan-Meier method, plotting survival probability (%) against time (days). The differences among the three groups were tested using the log-rank test and P-values (0.0107 by Log-Rank, 0.0241 by Wilcoxon) and showed that the correlation of *PTK6* mRNA levels and survival was statistically significant. High *PTK6* mRNA levels correlate with poor survival, while the low *PTK6* expressing group has the best survival.

## DISCUSSION

These studies are the first to report notable PTK6 expression in the normal human mammary gland. In contrast to previous reports utilizing immunohistochemistry (i.e. 24, 34), we employed a tissue array that was specifically designed to analyze gene expression in normal mammary gland tissue [32]. This array did not include adjacent tumor tissue with high PTK6 expression that could overwhelm or compete with a signal in the normal breast tissue. Early studies of PTK6/BRK expression in normal mammary gland tissue used immunoblotting, RNase protection, RT-PCR and qRT-PCR assays with RNA isolated from tissue obtained from reduction mammoplasty surgeries, and PTK6 protein and transcripts were not detected [2, 5] or detected at very low levels [3]. Without enrichment for epithelial cells, the epithelium may not have been well represented in surgical discard tissues that would also have included adipose, endothelial and connective tissue. While we have not detected *Ptk6* protein or transcripts in the normal mouse mammary gland [23, 35], there may be distinctions in expression between species, related to lifespan and exposure to stress. PTK6 can be induced by stress and DNA damage in the intestinal epithelium and nontransformed cells where it promotes apoptosis [36-38]. Recently, PTK6 was shown to be regulated by hypoxia-inducible factors 1α/2α and proposed to be a mediator of hypoxia-induced breast cancer progression [39]. Similar to findings in human breast cancer, we recently demonstrated that PTK6 is induced in mouse mammary gland tumors of different origins, including spontaneous tumors and ERBB2 (HER2) induced tumors [23].

We detected total PTK6 protein in the cytoplasm and nuclei of epithelia in normal human mammary glands (Fig. 2). However, we did not detect PTK6 phosphorylated at Y342. PTK6 tyrosine residue 342 corresponds to tyrosine residue 419 in human SRC (416 in chicken), and phosphorylation of these residues within the kinase catalytic domain corresponds with increased kinase activity [31, 40]. Absence of detectable P-Y342 suggests that PTK6 functions in the normal mammary gland could be kinase-independent. Studies have shown that PTK6 may have both kinase-dependent and -independent scaffold/adaptor functions [41, 42]. Nuclear targeted PTK6 inhibited β-catenin regulated transcription in a kinase-independent manner [42].

Gene expression studies have led to the identification of different molecular subtypes of invasive breast cancer, including the basal-like triple negative (ER−/PR−/HER2−), HER2 overexpressing, and luminal A (ER+ and/or PR+/HER2−) and luminal B (ER+ and/or PR+/HER2+) subtypes [43, 44]. We detected high total PTK6 protein immunoreactivity (scored as 2 - 3+) in all subtypes; over 80% of luminal A and luminal B patients exhibited strong PTK6 protein expression (Table 3). While no PTK6 P-Y342 was detected in the normal mammary gland samples, we detected membrane associated active P-Y342 PTK6 in all tumor subtypes, with the highest percentages of strong positives in the triple negative (25%) and HER2 overexpressing (25%) subtypes (Table 3).

**Table 3:** Expression and activation of PTK6 in different human breast tumor subtypes Data are summarized for each patient; # represents the number of patients in each group. Triple negative: ER/PR negative, with absent or low HER2 (0 - 2+); HER2: ER/PR negative with high HER2 (3+); Luminal A: ER/PR positive, with low-grade/proliferation; Luminal B: ER/PR positive with high-grade/proliferation, some may have high HER2 (3+). P-PTK6 represents P-Y342 PTK6

	#	PTK6 0+	PTK6 1+	PTK6 2-3+	P-PTK6 0+	P-PTK6 1+	P-PTK6 2-3+
Triple Negative	16	0 (0%)	6 (37.5%)	10 (62.5%)	6 (37.5%)	6 (37.5%)	4 (25%)
HER2	4	0 (0%)	1 (25%)	3 (75%)	0 (0%)	3 (75%)	1 (25%)
Luminal A	18	1 (5.6%)	2 (11.1%)	15 (83.3%)	11 (61.1%)	4 (22.2%)	3 (16.7%)
Luminal B	6	0 (0%)	1 (16.7%)	5 (83.3%)	2 (33.3%)	3 (50%)	1 (16.7%)

Striking localization of PTK6 P-Y342 at the plasma membrane was detected in IDC, the most common type of breast cancer, as well as in DCIS, the most common non-invasive breast cancer (Fig. 3). In contrast, total PTK6 and PTK6 P-Y342 were localized in nuclei in high-grade LCIS, a premalignant condition that indicates risk for developing invasive breast cancer (Fig. 4A). Active PTK6 P-Y342 was difficult to detect in a low-grade LCIS (Fig. 4A). In patient cores containing both LCIS and DCIS, active PTK6 P-Y342 was detected in nuclei and at the membrane respectively (Fig. 4B). No membrane associated active PTK6 P-Y342 was detected in apocrine metaplasia, a common benign condition (Fig. 4A). Interestingly, in breast tumors expressing high levels of PTK6 P-Y342, luminal A, B and HER2 subtypes contain both IDC and DCIS or LCIS. In contrast 3/4 triple negative tumors were classified as high-grade IDC (Fig 3, IDC-HG), suggesting a possible correlation of high levels of membrane-associated PTK6 P-Y342 with poor prognosis. Although the sample size was limited, these data suggest that PTK6 activation at the membrane as measured by P-Y342 may be most apparent in invasive breast cancers.

Increased *PTK6* mRNA expression relative to normal tissue was previously reported to be significant independent of tumor subtype in invasive ductal and invasive lobular carcinoma in the TCGA breast dataset [39]. Irie and colleagues also found increased *PTK6* expression in all breast cancer subtypes except basal when they analyzed three different data sets (Van't Veer, Hu, and Lu) [21]. Coamplification and overexpression of *PTK6* and *ERBB2* was detected in a dataset of 113 Norwegian breast tumors [20]. Here we correlated high *PTK6* mRNA expression with poor survival outcome in the TCGA dataset (Fig. 6), complementing earlier studies demonstrating high *PTK6* expression has a negative impact on patient outcome in the Van't Veer and Wang datasets [21]. Cumulatively, these results suggest that *PTK6* expression levels can serve as a tool for predicting breast cancer survival when the status of PTK6 protein activation is unavailable. However, PTK6 activation and alterations in its intracellular localization may not directly correlate with high levels of total protein or mRNA expression. Further studies are required to determine if activation of PTK6 occurs preferentially in specific tumor subtypes and/or high-grade invasive tumors. Our data suggest that it will be important to take the activation status and intracellular localization of PTK6 into account to fully understand its contributions to invasive breast tumors and its potential as a therapeutic target.

## MATERIALS AND METHODS

### Cell Culture

The human embryonic kidney cell line HEK293, the human breast cancer cell lines MDA-MB-231, SK-BR-3, MCF-7, MDA-MB-453, BT-474, T-47D, and the immortalized human breast epithelial cell line MCF-10A were obtained from and validated by ATCC. With the exception of MCF-10A cells, all cell lines were cultured according to ATCC guidelines. MCF-10A cells were cultured in DMEM/F12 with 5% horse serum, 20 ng/ml EGF, 0.5 μg/ml hydrocortisone, 100 ng/ml cholera toxin, 10 μg/ml insulin and 1 × Penicillin-Streptomycin.

### Protein lysates and immunoblotting

Cells were harvested at 80% confluence and lysed in Triton X-100 buffer (20 mM Hepes, pH7.4, 1% Triton X-100, 150 mM NaCl, 1 mM EDTA, pH8.0, 1 mM EGTA, pH8.0, 10 mM Na-pyrophosphate, 100 mM NaF, 5 mM iodoacetic acid, 1 mM sodium vanadate, 0.2 mM PMSF and proteinase inhibitor cocktail tablet (Roche Diagnostic, Indianapolis, IN). Immunoblotting was performed as previously described [42].

### Antibodies

Human PTK6 C-18 (SC-1188) and human PTK6 G6 (SC-166171) were obtained from Santa Cruz Biotechnologies (Santa Cruz, CA); phospho-PTK6 Tyr-342 (P-Y342) (09-144) was purchased from Millipore (Billerica, MA), and rabbit IgG from Vector Laboratories (Burlingame, CA). Antibodies against β-actin (A-5441) and α-tubulin (T-9026) were from Sigma-Aldrich(St. Louis, MO).

### Real-time PCR and semi-quantitative PCR

Total RNA was isolated with TRIzol Reagent (Life Technologies, Grand Island, NY). After DNase (Promega, Madison, WI) digestion, cDNA was generated by reverse-transcription with iScript cDNA Synthesis kit (Bio-Rad, Hercules, CA). Real-time PCR was performed with primers that target *PTK6* exon 2 which is specific for full length *PTK6*. Human cyclophilin was used as internal control. PCR reactions were set up in triplicate with iQ SYBR Green Supermix (Bio-Rad) and run in the MyiQ single-color real-time PCR detection system (Bio-Rad). Primer sequences: PTK6 X2-F: 5' - CGG AAC CGT GGT TCT TTG - 3'; PTK6 X2-R: 5' - ACT CGG CTT CTC CGC TGA C - 3'; Cyclophilin-F: 5' - GCA GAC AAG GTC CCA AAG ACA G - 3'; Cyclophilin-R: 5' - CAC CCT GAC ACA TAA ACC CTG G - 3'. Data were analyzed with Bio-Rad iQ5 software and processed with Microsoft Excel. Semi-quantitative PCR was performed in a BioRad MyCycler PCR machine with primers flanking exon 2 of human *PTK6*, which yield a 407 bp fragment for full length *PTK6* and a 285 bp fragment for *ALT-PTK6*. Primer sequences: PTK6-Del-F: 5' – GCT ATG TGC CCC ACA ACT ACC – 3'; PTK6-Del-R: 5' – CCT GCA GAG CGT GAA CTC C -3'.

### Immunohistochemistry and immunofluorescence

The Vectastain ABC kit (Vector Laboratories) and 3, 3'-Diaminobenzidine tetrahydrochloride tablets (Sigma-Aldrich) were used for immunohistochemistry. Antigen retrieval was done in sub-boiling 0.01 M sodium citrate solution for 20 minutes. Samples were incubated in 3% H_2_O_2_ prepared in methanol for 10 minutes to quench endogenous peroxidase, and then washed with PBS and blocked with serum before incubating with primary antibody at room temperature for 1 hour. As a control for specific staining, tissue sections were also stained with IgG antibodies of the same concentration as the primary antibody.

A peptide competition assay was performed to examine the specificity of the PTK6 C-18 antibody. PTK6 C-18 was pre-incubated with five-fold excess (by weight) of the peptide antigen used for raising the antibody (SC-1188p, Santa Cruz Biotechnologies) in PBS for 2 hours at room temperature, then incubated at room temperature for 1 hour as primary antibody on the tissue. Antibody without blocking peptide was also incubated at room temperature for 2 hours before applying to the tissue as a control.

For immunofluorescence, slides were blocked with 3% BSA in TNT buffer (0.1 M Tris-HCl, pH7.5, 150 mM NaCl, 0.05% Tween 20) for one hour and incubated with primary antibodies at 4°C overnight. After washing in TNT buffer, slides were incubated with biotinylated anti-rabbit secondary antibodies and then fluorescein isothiocyanate (FITC)-conjugated avidin (Vector Laboratories). Slides were mounted with mounting medium containing DAPI from Vector Laboratories.

Slides were examined and imaged on a Zeiss LSM 700 confocal microscope using the manufacturer's imaging software ZEN (ZEISS Efficient Navigation). Exposure times for FITC were fixed for each antibody across the samples, while times for DAPI varied depending on the signal strength.

### Patient samples and tissue arrays

The normal human mammary gland TMA has been described [32]. Samples on the TMA were obtained from women between 18 and 45 years of age who underwent a reduction mammoplasty or breast biopsy with benign findings at the University of Illinois Hospital. Seventy-six 2.0 mm diameter cores from 27 patients with benign findings were analyzed.

Paraffin-embedded tissues were obtained from patients diagnosed with breast cancer between 18 to 45 years of age from the University of Illinois at Chicago Hospital, Northwestern Memorial Hospital (Chicago) and Allina Hospitals and Clinics (Minneapolis, MN) and processed as previously described [32]. The breast cancer TMA was constructed with three 2.0 mm diameter cores placed adjacent to each other resulting in 131 cores from 45 patients on the TMA. These studies were approved by the Institutional Review Boards of each institution.

### Pathology analysis

The tumors were first analyzed by examining morphology of hematoxylin and eosin stained tissue microarrays to identify in situ and invasive cancers. In situ tumors were separated into low and high-grades using the Van Nuys system, while invasive mammary carcinomas were stratified into grades 1-3 using the Nottingham combined histologic grading system. [45-47]. Based on the estrogen and progesterone hormone receptors and ERBB2 (HER2) phenotype by immunohistochemistry, invasive mammary carcinomas were also categorized as belonging to the Luminal A (hormone receptor positivity with low tumor grade and proliferation), Luminal B (hormone receptor positivity with high tumor grade and proliferation) and HER2-enriched groups (negative hormone receptors and HER2 3+ positivity by immunohistochemistry) (see table 3) [48].

### Survival analysis

Data from TCGA Breast (The Cancer Genome Atlas, Office of Cancer Genomics, National Cancer Institute, National Institutes of Health, Bethesda, MD 20892) [33] were downloaded from the Oncomine database and included 532 invasive breast carcinoma, 61 paired with normal breast tissue and 3 paired metastatic samples. This dataset consists of Level 2 (processed) data from the TCGA data portal (http://tcga-data.nci.nih.gov/tcga/), and contained 447 breast samples with associated survival information. Patients were arbitrarily categorized into “high”, “medium” and “low” groups according to their *PTK6* mRNA expression levels. These categories and the numbers of patients in each group are shown in Figure 6. The Kaplan-Meier curve was plotted using SAS 9.2 and the log-rank test and P-value were used to determine significance.

## SUPPLEMENTARY MATERIAL FIGURES


