# Supplementary material for: PTK6/BRK is expressed in the normal mammary gland and activated at the plasma membrane in breast tumors
Source: Oncotarget. 2014 Jun 30;5(15):6038–48. doi: 10.18632/oncotarget.2153 (PMC4171611; doi:10.18632/oncotarget.2153)
Supplement: Supplementary file 1 [file oncotarget-05-6038-s001.pdf]

PTK6/BRK is expressed in the normal mammary gland and activated at the plasma membrane in breast tumors

Supplementary Material

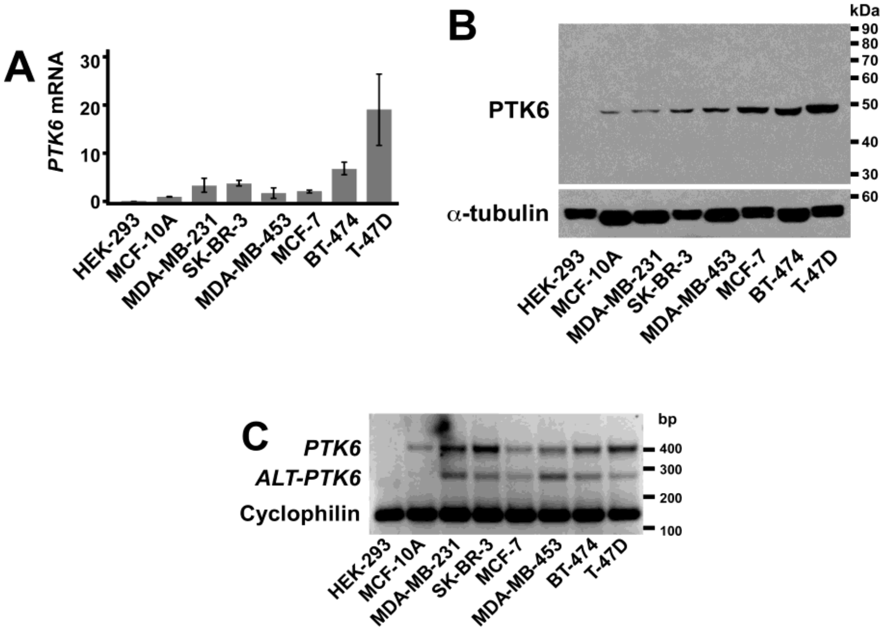

Supplementary Figure 1: *PTK6* mRNA and protein expression in a non-transformed breast epithelial cell line and multiple breast cancer cell lines. Endogenous PTK6 expression was analyzed by real-time PCR and immunoblotting. (A) To detect the mRNA levels of endogenous *PTK6*, total mRNA isolated from several breast cancer cell lines and the immortalized MCF-10A cell line was reverse-transcribed into cDNA. Quantitative real-time PCR was performed using primers that target exon 2, which is specific to the full length *PTK6*. *PTK6* mRNA levels were normalized with cyclophilin mRNA levels, and MCF-10A was set as standard for the comparison. HEK293 cells do not express *PTK6* and were used as a negative control. (B) Immunoblotting with total cell lysates was performed to detect PTK6 protein; α-tubulin was used as loading control. (C) Full length *PTK6* (407 bp) and *ALT-PTK6* (285 bp) were amplified with a set of primers flanking human *PTK6* exon 2. Cyclophilin was amplified as a control.

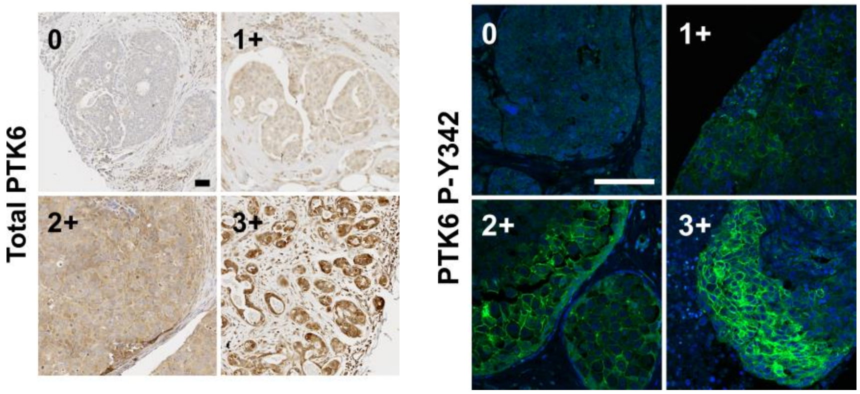

Supplementary Figure 2: Demonstration of the 0 to 3+ scoring scale used to evaluate PTK6 expression levels. Examples of different total PTK6 (left panels) and active PTK6 P-Y342 (right panels) expression levels are shown. The scale bars represent 50 μm.
